# Multi-View-Based Pose Estimation and Its Applications on Intelligent Manufacturing

**DOI:** 10.3390/s20185072

**Published:** 2020-09-07

**Authors:** Haiwei Yang, Peilin Jiang, Fei Wang

**Affiliations:** School of Electronic and Information Engineering, Xi’an Jiaotong University, Xi’an 710049, China; yanghw.2005@stu.xjtu.edu.cn (H.Y.); pljiang@mail.xjtu.edu.cn (P.J.)

**Keywords:** pose estimation, multiple views, intelligent manufacturing

## Abstract

Pose estimation is a typical problem in the field of image processing, the purpose of which is to compare or fuse images acquired under different conditions. In recent years, many studies have focused on pose estimation algorithms, but so far there are still many challenges, such as efficiency, complexity and accuracy for various targets and conditions, in the field of algorithm research and practical applications. In this paper, a multi-view-based pose estimation method is proposed. This method can solve the pose estimation problem effectively for large-scale targets and achieve good performance accuracy and stability. Compared with existing methods, this method uses different views (positions and angles), each of which only observes some features of large-size parts, to estimate the six-degree-of-freedom pose of the entire large-size parts. Experimental results demonstrate that the accurate six-degree-of-freedom pose for different targets can be obtained by the proposed method which plays an important role in many actual production lines. What is more, a new visual guidance system, applied into intelligent manufacturing, is presented based on this method. The new visual guidance system has been widely used in automobile manufacturing with high accuracy and efficiency but low cost.

## 1. Introduction

Pose estimation has been widely used in aerospace [1,2], unmanned driving [3], augmented reality [4,5], intelligent robots [6,7], thermal analysis [8,9,10], and automobile manufacturing [11,12]. It is a vital research direction in the field of computer vision. Now, six-degree-of-freedom pose estimation (6D pose estimation) is the dominante trend. It refers to the technique of acquiring RGB image or depth image of a target and using the image to estimate three position parameters and three orientation parameters of the object in a specific coordinate system.

6D pose estimation has played a prominant part in some typical fields. For example, in the augmented reality, it is used to perceive the real world, that is, to estimate the position and posture of objects in the real world, so that the information of the virtual world can be reasonably superimposed on the real world. In the aerospace field, it is also widely used. Especially at the space rendezvous and docking, in order to complete the docking work successfully, the relative attitude between the docking spacecraft and the target spacecraft needs to be adjusted, using the pose estimation technology. Besides, 6D pose estimation technology also can help the removal of space debris. It is applied when the satellite manipulator estimates the pose of space debris and captures the debris. In the unmanned driving, 6D pose estimation technology is used to estimate the relative pose between unmanned vehicles and people, which provides a guarantee for the safety of unmanned driving. In the field of intelligent robots, visual simultaneous locating and mapping (vSLAM) uses 6D pose estimation technology to achieve intelligent robot path planning. In the automobile manufacturing, 6D pose estimation technology is used to implement tasks such as intelligent grasping, painting and welding.

Although 6D pose estimation technology has been widely used in many fields, the applications of 6D pose estimation technology still face many difficulties, such as, the occlusion problem, the cluster problem, the flexibility for different objects, the dependence on the data set, and the low measurement precision. These difficulties put forward higher requirements on the accuracy and robustness of 6D pose estimation algorithm. With the computing power constantly increasing and achievements in deep learning emerging, the 6D pose estimation technology has gained many exciting developments. However, most of them aim at special or small targets, limited by the camera’s filed of view. Few studies are focused on large-scale or different-size targets, although there are many urgent needs for accurate and efficient 6D pose estimation of large-scale targets. For example, in the intelligent manufacturing of automobile, there are a lot of large-size parts to be handled. Therefore, it is necessary to determine their positions and orientations, which are relative to cooresponding robots, in order to achieve intelligent grasping, transporting, welding, spraying and so on. To sum up, the traditonal 6D pose estimation technology needs further exploration and development, in order to meet the demand of intelligent manufacturing.

With the development of technology, industrial robots have been widely used in industrial production. In traditional way, the industrial robots are taught to finish so many grasping tasks, in which each part is fixed tightly by complex fixture. The disadvantages are obvious. Firstly, this process requires manual involvement because it requires parts to be accurately fixed before being grasped. Secondly, the process of placing parts usually takes a long time, which affects the efficiency of production. Thirdly, fixtures will be worn out during the production cycle, which will cause deviations in the fixed position of the part and then lead to the failure of the grasping task. Fourthly, it is very inefficient and complicated to re-customize fixtures when new parts are also needed to be accurately fixed. Therefore, industrial manufacturing has a very urgent need for intelligent technology which can measure the pose of each part and guide the robot to automatically handle them. In industrial manufacturing, the requirement of grasping accuracy is usually at the millimeter level [13]. There are some real-time pose measurement devices that can be used for guiding robot, such as the indoor global positioning system (iGPS) [14,15], and the workspace measuring and positioning system (wMPS) [16,17]. iGPS and wMPS have the characteristic of high precision. However, these equipments are large and expensive, not suitable for general industrial applications. Vision system for robot guidance has been used in automotive industry since the end of 20th century. In recent years, vision measurement technology has been developed rapidly. The robotic visual system, which uses vision measurement technology for robot guiding, has been applied extensively in the aerospace, aircraft and automotive manufacturing industries [18,19,20,21,22].

There are several different vision measurement solutions such as monocular vision [23], binocular vision [24], structured light method [25] and multiple-sensor method [26]. In general, the three dimensional information about the part cannot be obtained directly with monocular vision [27]. The structured light method needs the assistance of laser, but it is limited by laser triangular measurement principle. So it can’t be used to estimate the pose of large-size parts [28]. In Reference [29], a monocular-based 6-DOF pose estimation technology is proposed for robotic intelligent grasping systems. It can estimate the pose of large-size parts through the camera movement. However, this movement is restricted to translation only, which limits their applications. What’s more, this method cannot obtain an accurate initial value of the part pose, which makes it possible to fall into a local minimum in subsequent nonlinear optimization.

In this paper, we focus on the applications of multi-view-based pose estimation. The ultimate goal of this paper is to seek a 6D pose estimation method with high accuracy, high efficiency but low cost, so that it can be widely used in the field of intelligent manufacturing. The multi-view-based pose estimation method, proposed by us, can handle the 6D pose estimation of large-scale targets. Its applications on intelligent manufacturing and are explored deeply. Compared with existing mainstream methods or applications in industrial manufacturing, our multi-view-based 6D pose estimation method can be directly used in many applications, due to its efficiency for different industrial parts.

The contributions of this work are three aspects: First, we setup a visual guidance system for robotic intelligent grasping, which can estimate the 6-degree-of-freedom (6-DOF) pose of the part and guide the robot to grasp it accurately. Second, we introduce a fast hand-eye calibration method, which can quickly calibrate the relative transformation between the camera and the robot end-effector. Third, we propose a multi-view-based 6-degree-of-freedom pose estimation method for large size parts. Compared with existing methods, the present method can estimate the 6-DOF pose of large-size parts through capturing large-size parts from several different positions and orientations. Specially, we use binocular reconstruction technology to calculate the initial value of the pose before nonlinear optimization, to ensure the validity and accuracy of the final pose. Experimental results demonstrate the proposed method can obtain accurate 6-DOF pose. Moreover the visual guidance system can accomplish intelligent grasping tasks.

## 2. Methods

### 2.1. Visual Guidance System

In automotive manufacturing, different parts or workpieces are assembled through robots. Due to the lack or abrasion of fixtures which are used for positioning parts, the position and orientation of these part are not fixed before the robot grasps the part. Therefore, in order to guide the robot, it is necessary to introduce a visual guidance system to measure the 6-degree-of-freedom pose between the parts and the robot, which can ensure the grasping accuracy and the production efficiency. Specially, we use binocular vision system to enhance stability.

**Figure 1:**
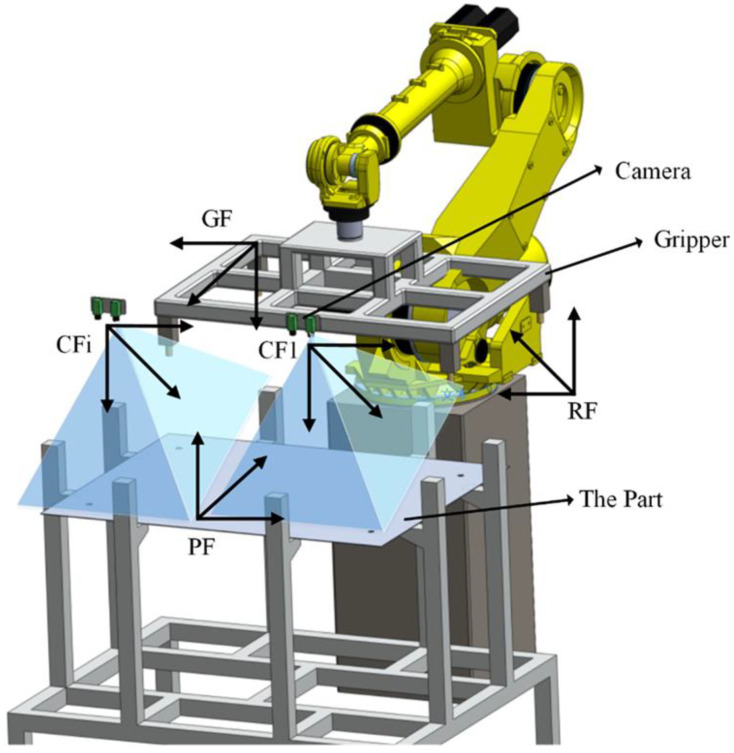
The coordinate systems of the robotic intelligent grasping system consist of robot frame (RF), parts frame (PF), robot gripper frame (GF) and camera frame (CF).

As shown in Figure 1, before the robot grasps the part, it moves the camera to measure several feature points on it, and then determines the pose of the part. Parts vary in size and differ in shape, as small-sized parts may be less than 0.5 m × 0.5 m, while large-sized parts may be larger than 1.5 m × 1.5 m. For smaller sized parts, all feature points are included in the camera’s field of view. In this case, the robot only needs to move and observe at one position. However, for larger parts, all feature points cannot be included in one camera’s field of view. To ensure that all feature points are measured, the robot needs to be moved to multiple positions and capture different images of the part through multi-views. The coordinate systems of the robotic intelligent grasping system consist of robot frame (RF), parts frame (PF), robot gripper frame (GF) and camera frame (CF).

In this system, camera is fixed on the end of robot arm and can be moved to different measurement positions along with the robot. The camera frame at the first measurement position, is called the first camera frame (CF1), which is taken as the reference frame. Due to the high precision of industrial machine, the parts frame (PF) can be obtained from their models in computer-aided design (CAD) software. The visual guidance system is described in detail as follows:

(1) The robot measurement path is determined by robot teaching, with which the robot brings the camera to measure the feature points on the part. Then the part pose ${}_{PF}^{CF1}T$ is determined, which is the transformation from parts frame PF to the first camera frame CF1.

(2) When the target is rest on the initial position, the grasping path is obtained through robot teaching. As shown in Figure 1, the transformation ${}_{PF0}^{CF1}T$, from part frame at initial position PF0 to the camera frame at the first measurement position CF1, can be given as follows:

(1) $${}_{PF0}^{CF1}T^{-1}={}_{CF1}^{PF0}T={}_{GF0}^{PF0}T\times {}_{RF}^{GF0}T\times {}_{CF1}^{RF}T,$$

where ${}_{CF1}^{RF}T$ is the transformation from CF1 to RF; ${}_{RF}^{GF0}T$ is the transformation from RF to GF0; ${}_{GF0}^{PF0}T$ is the transformation from GF0 to the initial part frame PF0, where GF0 is the robot tool frame when the robot grasp the part at the initial position.

When the robot grasps the part, the relative pose between gripper and the part is fixed, which means

(2) $${}_{GF}^{PF}T={}_{GF0}^{PF0}T={}_{GFi}^{PFi}T.$$

Therefore, the transformation can be reformated as:

(3) $${}_{PF0}^{CF1}T^{-1}={}_{CF1}^{PF0}T={}_{GF}^{PF}T\times {}_{RF}^{GF0}T\times {}_{CF1}^{RF}T.$$

When the part produces an offset relative to its initial position, the visual system needs to measure the pose of the part at current position. For small-size part and small offset, the camera can also work at the original measurement position. However, for large-size part or large offset, it does not work because current part has been out of the camera’s field of view. To solve this problem, we manipulate the robot moving to several positions, in order to make the camera measure current part from different views (positions and orientations). At the *i*-th view, the pose, from current part to the camera ${}_{PFi}^{CF1}T$, can be given as follow:

(4) $${}_{PFi}^{CF1}T^{-1}={}_{CF1}^{PFi}T={}_{GF}^{PF}T\times {}_{RF}^{GFi}T\times {}_{CF1}^{RF}T,$$

where ${}_{RF}^{GFi}T$ is the transformation from RF to GFi, and GFi is the robot gripper frame at *i*-th measurement position.

Due to the high accuracy of the robot, the transform relationship between CF1 and RF can be regarded as fixed.

(3) In the robot control system, the pose adjustment can be realized by frame offset. The robot can accurately grasp current frame, through changing the transform relationship between RF and GF with an offset:

(5) $${}_{Rf}^{GFi}T=\Delta_{RF}^{GF}T\times {}_{RF}^{GF0}T,$$

where $\Delta_{RF}^{GF}T$ is the frame offset transformation to be solved.

(4) Combining the above equations, the frame offset transformation is estimated as:

(6) $$\Delta_{RF}^{GF}T={}_{GF}^{PF}T^{-1}\times {}_{PFi}^{CF1}T^{-1}\times {}_{PF0}^{CF1}T\times {}_{GF}^{PF}T,$$

where ${}_{PF0}^{CF1}T$ and ${}_{PFi}^{CF1}T$ are the pose from part frame to the camera frame at initial position and *i*-th measurement position.

Finally, we can calculate the base frame offset transformation matrix, with which the robot could adjust its grasping path to achieve intelligent grasping.

### 2.2. Hand-Eye Calibration

Hand-eye calibration is to determine the transformation relationship between the camera frame CF and the gripper frame GF. In this paper, a chessboard is used for calibration (called calibration board).

**Figure 2:**
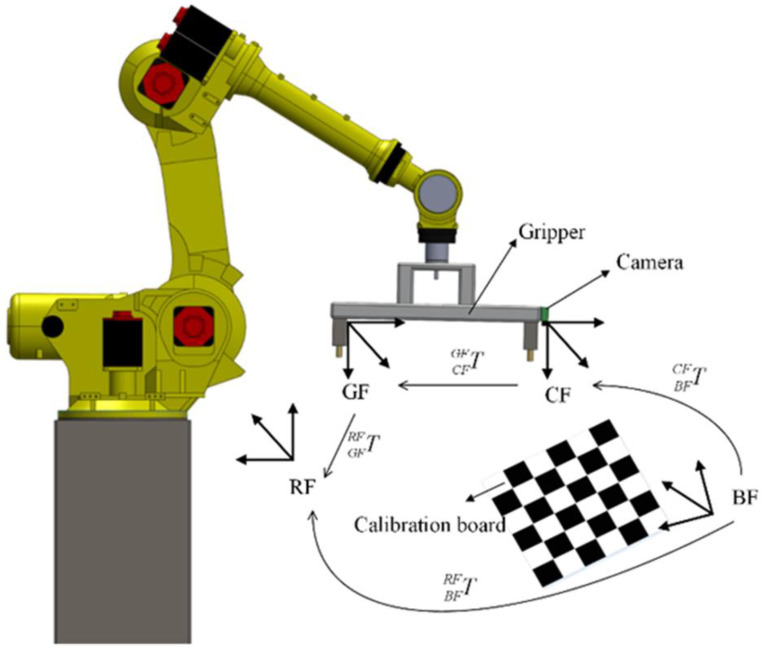
Hand-eye calibration is to determine the transformation relationship between CF and GF.

As shown in Figure 2, the chessboard is placed in the camera’s field of view, and the transformation relationship ${}_{BF}^{CF}T$ between calibration board frame (BF) and camera frame CF can be obtained by detecting the corner points on the calibration board. By obtaining the position of the corner points of the calibration board in RF, the transformation relationship ${}_{BF}^{RF}T$ between RF and calibration board frame can be calculated. Through the robot teaching device, the robot pose at current position can be achieved. Then the transformation ${}_{CF}^{GF}T$ can be calculated as follows:

(7) $${}_{CF}^{GF}T={}_{GF}^{RF}T^{-1}\times {}_{BF}^{RF}T\times {}_{BF}^{CF}T^{-1}.$$

Of course, we can also adopt other hand-eye calibration methods, such as eye-on-hand calibration method, in which the robot drives the camera to move and takes images of calibration borad at different positions and angles. In another way, the relationship between camera, calibration borad and the robot is established through high-precision measurement equipments (such as theodolite, laser tracker).

### 2.3. Multi-View-Based Pose Estimation

**Figure 3:**
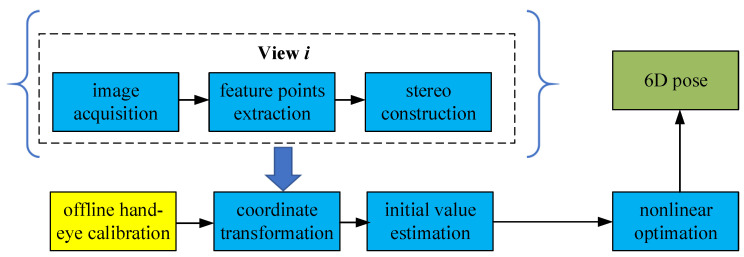
The framework of the multi-view-based 6D pose estimation method.

The multi-view-based 6-DOF pose estimation method is proposed for the pose estimation of large-size parts. In this situation, the feature points have a wider distribution and the camera can not see all the feature points in one single view. The camera, along with the robot, has to move to several different views to capture all the feature points. The framework of the multi-view-based 6D pose estimation method can be seen in Figure 3. The method begins with feature points extraction and stereo construction from each view. The final 6D pose is estimated after coordinate transformation, initial value estimation and nonlinear optimization.

In each view, we extract the image points by image processing methods. First, automatic image thresholding [30] is performed to separate the pixels into foreground and background. Second, the candidate objects (ellipses) are selected with their areas and shapes. Third, the coordinates of feature points are computed using Hough transform [31]. The corresponding 3D coordinates of these feature points can be achieved through stereo construction.

Based on camera perspective projection model, the relationship between the coordinate of the camera points $P_{C}$ and the image points $P_{I}$ are given as follows:

(8) $$\left\{\begin{array}{c} Z_{l}P_{Il}=A_{l}P_{Cl}\\ Z_{r}P_{Ir}=A_{r}P_{Cr}, \end{array}\right.$$

where $Z_{l}$ and $Z_{r}$ are the depth factors. $P_{Cl}={\left[x_{Cl},y_{Cl},z_{Cl},1\right]}^{T}$ and $P_{Cr}={\left[x_{Cr},y_{Cr},z_{Cr},1\right]}^{T}$ are the homogeneous coordinates of a spatial point in left camera frame and right camera frame separately. $P_{Il}={\left[u_{l},v_{l},1\right]}^{T}$ and $P_{Ir}={\left[u_{r},v_{r},1\right]}^{T}$ are the corresponding coordinates in image frame. $A_{l}$ and $A_{r}$ are the left and right camera intrinsic parameters, which can be calculated from the camera calibration method [32], which are formulated as:

$$A_{l}=\left[\begin{array}{cccc} f_{xl} & 0 & u_{l} & 0\\ 0 & f_{yl} & v_{l} & 0\\ 0 & 0 & 1 & 0 \end{array}\right],\;A_{r}=\left[\begin{array}{cccc} f_{xr} & 0 & u_{r} & 0\\ 0 & f_{yr} & v_{r} & 0\\ 0 & 0 & 1 & 0 \end{array}\right],$$

The coordinates $P_{Cl}$ and $P_{Cr}$ have the relationship:

(9) $$P_{Cr}={}_{l}^{r}T\times P_{Cl},$$

where ${}_{l}^{r}T=\left[\begin{array}{cc} {}_{l}^{r}R & {}_{l}^{r}t\\ 0 & 1 \end{array}\right]$ is the transformation from left camera frame to right camera frame, which can be computed by stereo camera calibration.

Assuming that there are M(M≥1) views and $n_{i}$ feature points for each view. Therefore, the total number of feature points for all views is

$$N=\sum_{i=1}^{M}n_{i}$$

Let $P_{j}^{i},\;1\leq j\leq n_{i},1\leq i\leq M$ represents the coordinate of *j*-th feature point at *i*-th view. We need to transform coordinates of feature points according to the transformation between CF1 and CFi. The transformation ${}_{CF1}^{CFi}T$ can be expressed as follows:

(10) $${}_{CF1}^{CFi}T={}_{CFi}^{GFi}T^{-1}\times {}_{GF1}^{GFi}T\times {}_{CF1}^{GF1}T={}_{CFi}^{GFi}T^{-1}\times {}_{GFi}^{RF}T^{-1}\times {}_{GF1}^{RF}T\times {}_{CF1}^{GF1}T,$$

where ${}_{CFi}^{GFi}T$ is the transformation from CFi to GFi.

Because the camera is fixed on the end of the robot, the transformation from camera frame to robot frame is determined, which means ${}_{CFi}^{GFi}T={}_{CF1}^{GF1}T={}_{CF}^{GF}T$. In this way, the above equation can be reformulated as:

(11) $${}_{CF1}^{CFi}T={}_{CF}^{GF}T^{-1}\times {}_{GFi}^{RF}T^{-1}\times {}_{GF1}^{RF}T\times {}_{CF}^{GF}T,$$

where ${}_{GFi}^{RF}T$ is the transformation from GFi to RF for *i*-th view, which can be obtained directly through robot controller.

Using the calculated transformation ${}_{CF1}^{CFi}T$, the coordinates of different views can be transformed to the first view:

(12) $${}^{T}P_{j}^{i}={}_{CF1}^{CFi}T^{-1}\times P_{j}^{i},$$

where ${}^{T}P_{j}^{i}$ is transformed coordinates in the first view.

When all the feature points are transformed to the first view, we can calculate an initial transformation from the part frame PF to the camera frame CF, which will be used in the following nonlinear optimization.

**Figure 4:**
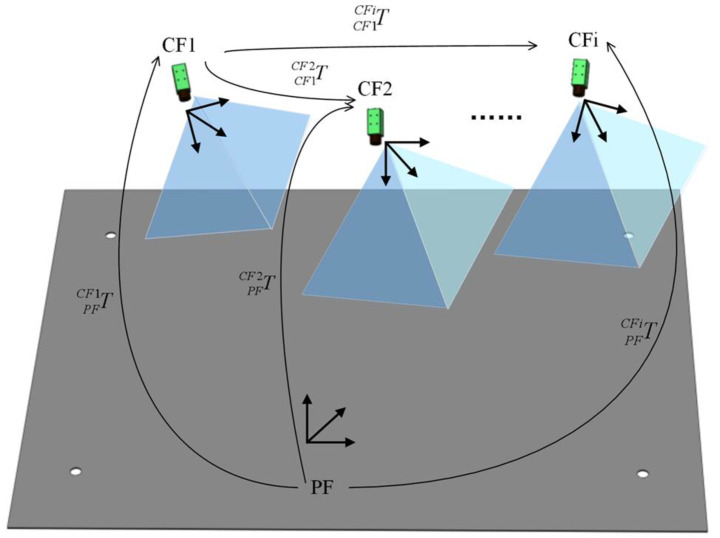
The transformation relationships between *PF* and *CF1* is known, and transformation relationships between *CF1* and *CFi* can be obtained. The transformation relationships between *PF* and *CFi* can be calculated.

In this section, the 6-degree-of-freedom pose of the part is obtained by nonlinear optimization based on the initial iteration value. As shown in Figure 4, the transformation relationship between PF and CFi can be given as follows:

(13) $${}_{PF}^{CFi}T={}_{CF1}^{CFi}T\times {}_{PF}^{CF1}T.$$

If the pose of the part relative to CF1 is known, the theoretical corresponding coordinate of feature points in image frame can be calculated based on camera perspective projection model. For *i*-th view, the theoretical corresponding coordinate of feature points in image frame can be calculated as follows:

(14) $$\begin{array}{cc} l_{i}\left[\begin{array}{c} {\hat{u}}_{j}^{i}\\ {\hat{v}}_{j}^{i}\\ 1 \end{array}\right] & =A_{l}\times {}_{PF}^{CFi}T\times P_{j}^{i}\\ \; & =A_{l}\times {}_{CF1}^{CFi}T\times {}_{PF}^{CF1}T\times P_{j}^{i}. \end{array}$$

Finally, the 6-DOF pose of the part relative to the first view can be estimated by minimizing the following objective funtion:

(15) $$\hat{T}=\arg min_{{}_{PF}^{CF1}T}\sum_{i=1}^{M}\sum_{j=1}^{n_{i}}\left[{\left(u_{j}^{i}-{\hat{u}}_{j}^{i}\right)}^{2}+{\left(v_{j}^{i}-{\hat{v}}_{j}^{i}\right)}^{2}\right],$$

where $\left(u_{j}^{i},v_{j}^{i}\right)$ the coordinates in image frame of *j*-th feature point at *i*-th view, and $\left({\hat{u}}_{j}^{i},{\hat{v}}_{j}^{i}\right)$ is the corresponding coordinates transformed to the image frame. Especially, when the number of views is 1 (M=1), the objective function becomes:

(16) $$\hat{T}=\arg min_{{}_{PF}^{CF1}T}\sum_{j=1}^{N}\left[{\left(u_{j}-{\hat{u}}_{j}\right)}^{2}+{\left(v_{j}-{\hat{v}}_{j}\right)}^{2}\right],$$

which is the traditional form of single-view pose estimation. In this sense, our method has universal applicability, and single-view pose estimation can be considered as a special form of our proposed multi-view-based pose estimation.

Equation (15) is derived from multi-view-based pose estimation problem of a moved camera. The formula is the sum of distance between the coordinates of the image point under current view and the coordinates of the projected image point transformed into this view. The transformation, which transform the image point from one view to another, plays an important role. In this sense, as long as the transformation relationship between the camera coordinate systems can be obtained, the formula can estimate the pose, whether it is one camera with multi-views, or one-shot with multi-cameras. From this side, it also illustrates the general adaptability and application potential of the formula we derived.

## 3. Experiments and Discussion

In this paper, we propose a multi-view-based pose estimation method and apply it in industrial manufacturing. In this experimental section, we verify the effectiveness of the proposed method and evaluate its role in actual production line from three aspects. First, simulation analysis is carried out to analyse its performance with different factors. Second, a verification system is built in laboratory, in order to evaluate the proposed visual guidance system. Third, the intelligent grasping system is applied into automobile manufacturing, which has played an important role in the actual production line.

### 3.1. Simulation Analysis

The accuracy of multi-view-based pose estimation method proposed in this paper is affected by many factors, such as the detection accuracy of feature points, the number of feature points and the number of views. We first design the simulation experiments with generated data, in order to further study the influence of these factors on the final results of multi-view-based pose estimation.

- Influence of feature point detection accuracy on multi-view-based pose estimation

**Figure 5:**
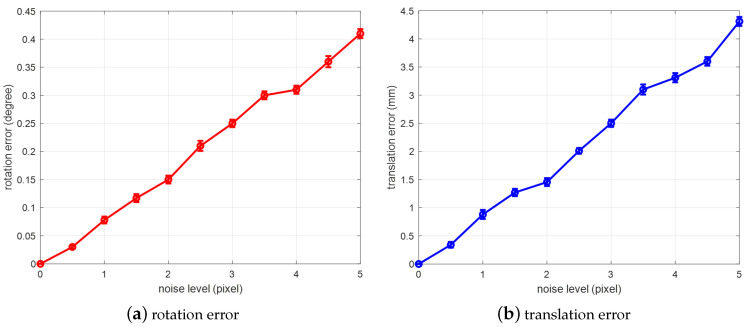
Influence of feature point detection accuracy on multi-view-based pose estimation: (**a**) rotation error; (**b**) translation error.

We set the number of feature points to 4 and the number of multi-views is 2. The 3D coordinates of each feature point in the workpiece coordinate system are adopted according to the CAD model of an automobile front floor. The ground-truth 2D coordinates in image frame is generated through camera perspective model with a ground-truth pose value. Then, based on the ground-truth coordinates, Gaussian noises with different levels are added to each feature point. The pose is estimated using the proposed multi-view-based pose estimation method. Finally, the deviations of rotation and translation are calculated, comparing the estimated pose and the ground-truth value. For each noise level, 2000 tests are conducted, and the average error is obtained. The result is shown in Figure 5:

It can be seen from the results that, as the noise level gradually increases, the deviation of the pose estimation also increases, and is approximately linear. When the noise level is 1 pixel, the rotation error is about 0.1°, with the translation error about 1 mm. This shows that the estimated pose is influenced by feature point detection accuracy. In practice, We should reduce the detection error of feature points by improving image quality or adopting special image processing algorithms

- Influence of number of feature points on multi-view-based pose estimation

**Figure 6:**
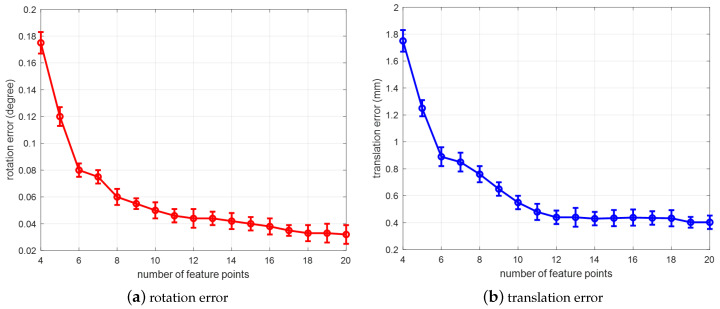
Influence of number of feature points on multi-view-based pose estimation: (**a**) rotation error; (**b**) translation error.

In order to determine the influence of number of feature points on multi-view-based pose estimation, we set the number of feature points growth from 4 to 20. In each group, the number of multi-views is fixed at 2 and the Gaussian noise is set to 1 pixel. Then we randomly generate a corresponding number of feature points within a certain range. In each test, we perform 2000 times with the added Gaussian noise level, while we calculate the average error of rotation and translation between the estimated pose and the ground-truth value. The result is shown in Figure 6:

As shown in the simulation results, when the number of feature points increases, the pose deviation decreases rapidly; when the number of feature points increases to a certain number (>16), the deviation of the pose estimation tends to be stable and basically does not change. Experiments show that increasing the number of feature points within a certain range can improve the accuracy of pose estimation. Therefore, we should select as many reliable feature points as possible.

- Influence of number of multi-views on multi-view-based pose estimation

**Figure 7:**
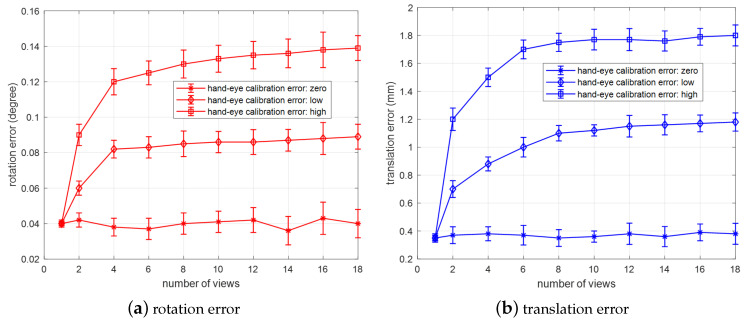
Influence of number of multi-views on multi-view-based pose estimation: (**a**) rotation error; (**b**) translation error.

In order to study the influence of number of multi-views on multi-view-based pose estimation, we conduct three different simulation experiments. One is to not consider the hand-eye calibration error (zero), the other two are to consider the hand-eye calibration errors. In this situation, we first add a smaller hand-eye calibration error (hand-eye calibration error: low-level), and then a larger hand-eye calibration error (hand-eye calibration error: high-level) is added between different views. In the simulation, the number of views is increased from 1 to 20, the number of feature feature points is 18, and Gaussian noise level is 1 pixel. Simulation experiments are conducted 2000 times with the noise level at each number of multi-views, for three different hand-eye calibration error (zero, low-level, high-level). The average error of rotation and translation between the calculated pose and the ground-truth value is analyzed. The final result is shown in Figure 7.

As shown in Figure 7, when the hand-eye calibration error is zero, the deviation of pose estimation does not increase with the growth of multi-views. Actually, the deviation basically remains unchanged, and is only affected by the accuracy of the feature point detection. However, when different levels of hand-eye calibration are introduced: As the number of multi-views increases, the pose estimation deviation gradually grows. The larger the hand-eye calibration error it has, the larger the estimated deviation it gains. This is because the error of hand-eye calibration will affect the solution of the objective function, resulting in deviation of pose estimation. However, when the views increase to a certain number (≥6), as the number of multi-views increases, the pose deviation tends to stabilize. This shows that the effect of hand-eye calibration errors is random. When there are many views, the offsets of the hand-eye calibration errors between different views are random and will be eliminated by an average way, to some extent. The simulation results show that when there are hand-eye calibration errors, the increase in the number of multi-views will affect pose estimation accuracy, resulting in poor accuracy. In practice, when facing large-size target, we have to use multi-views to estimate its pose. At this time, it is necessary to control the error of hand-eye calibration as much as possible. Therefore, when applied, on the premise of being able to estimate the pose of the object, we should select as few views as possible. In particular, after the number of multi-views is increased to more than 6, the effect of increasing the number of multi-views on pose estimation accuracy tends to be stable, the translation error is less than 1.8 mm, and the rotation error is less than 0.14°. The proposed algorithm still has high accuracy.

From the above three simulations, the standard deviations of rotation error and translation error are about 0.01° and 0.1 mm, for all the conditions (noise level, number of feature points, number of views), which also shows our method has good stability.

Our method is aimed at the pose estimation problem of large size targets and its application in intelligent manufacturing. The similar method is in Reference [29], in which a monocular-based 6-DOF pose estimation technology is proposed for robotic intelligent grasping systems. It can estimate the pose of large-size parts through the camera movement. To verify the adaptability of our proposed method, we follow the settings and data in Reference [29], moving without rotation, and use the same comprehensive angle and position error calculated as:

(17) $$\left\{\begin{array}{c} dR=\sqrt{\Delta \alpha^{2}+\Delta \beta^{2}+\Delta \gamma^{2}}\\ dT=\sqrt{\Delta X^{2}+\Delta Y^{2}+\Delta Z^{2}}. \end{array}\right.$$

**Figure 8:**
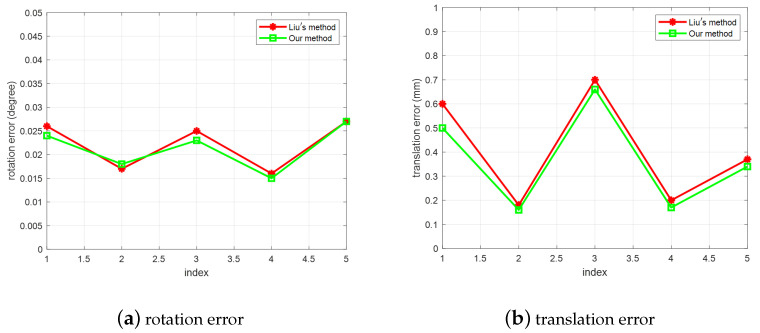
The comprehensive rotation and position errors of two methods: (**a**) rotation error; (**b**) translation error.

The estimation errors of the two methods are compared in Figure 8. We can see that there is no significant difference between Liu’s method and our proposed method, when limiting the camera movement to translation instead of rotation. However, In industrial applications, the parts have different situations and the camera should observe them from various views. If the camera movement is restricted to just translation, not rotation, the effectiveness of pose estimation method would be greatly reduced. Therefore, in the following experiments, only our proposed method can be applied, because the movements have both rotation and translation in automoble manufacturing.

### 3.2. Experimental Validation in Laboratory

**Figure 9:**
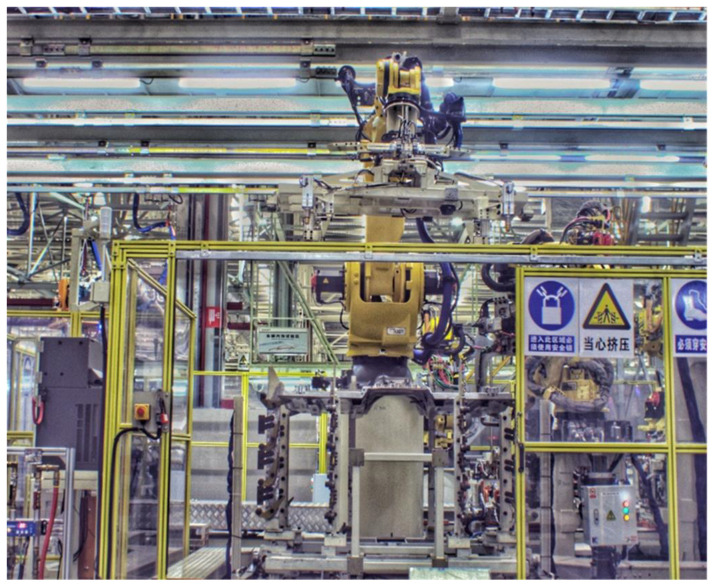
Experimental setup for robot intelligent grasping system. Robot at reset position.

**Figure 10:**
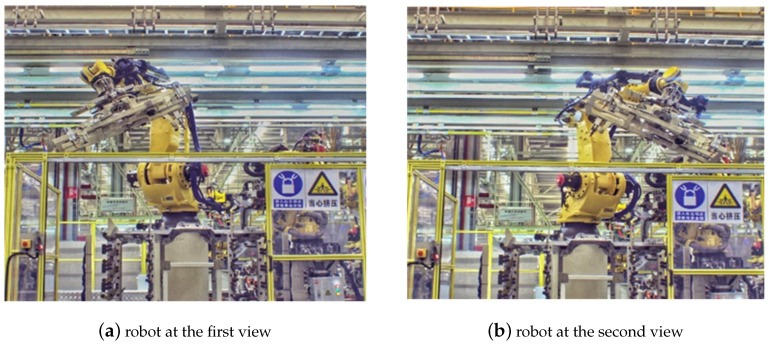
Experimental setup for robot intelligent grasping system: (**a**) Robot at the first view; (**b**) Robot at the second view.

**Figure 11:**
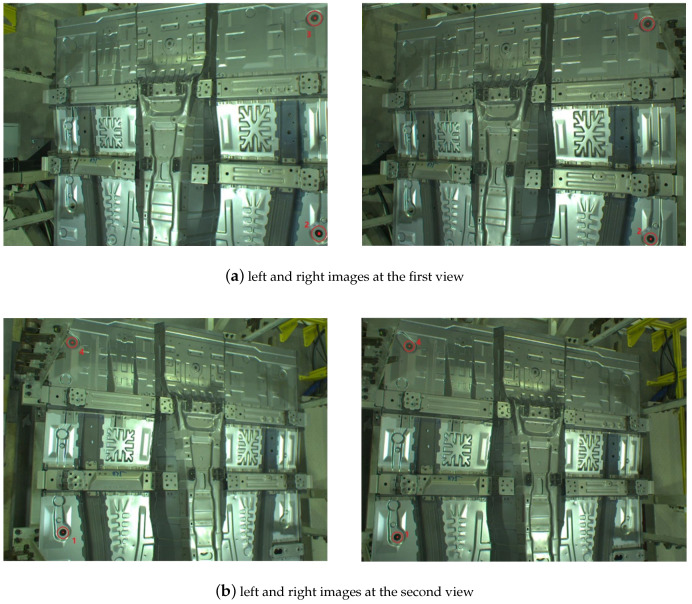
Two images (left camera and right camera) are captured at the first view (first row) and the second view (second row). Feature points 2 and 3 are measured at first view. Feature points 1 and 4 are measured at second view: (**a**) left image and right images at the first view; (**b**) left and right images at the second view.

In order to verify the effectiveness of the proposed multi-view-based pose estimation method, a robotic intelligent grasping system is setup and experimental validation is performed. As shown in Figure 9, the experimental setup consists of an industrial robot (Fanuc R-2000iA), two industrial cameras (Basler acA2040-35gc). Two cameras are mounted on the fixture of robot. A front floor of automobile is used as the test part. The floor part is a large-size automobile part and the cameras have to move to two different positions to cover it (Figure 10). There are four feature points, two of which are measured in each view (Figure 11).

The accuracy validation experiments are carried out on the experimental setup in Figure 9. After the hand-eye calibration, The front floor part is placed at several arbitrary positions. In each position (named as “Index”), the following two steps are carried out. First, the pose of the deviated floor part relative to the initial pose is measured by robot teaching (ground-truth value). Second, the robot brings the camera to capture the feature points through two views, and the pose is calculated with the proposed method (seen as computed value).

As shown in Table 1, the part (front floor) is conducted at 9 different positions (from Index 1 to 9). Compared with robot teaching, the root mean squared error for the angle and position is about 0.123° and 0.731 mm; furthermore, the standard deviations are about 0.053° and 0.220 mm. Due to the consistency error of the automobile body and parts, when grasping this workpiece, the required accuracy is usually 0.2° and 1.0 mm [13], which is also the usual accuracy of robot manual teaching. Therefore, it has reached the accuracy requirements of robot intelligent grasping system [29]. The robot grasping achieves good performance using the pose estimated by the proposed method.

### 3.3. Applications in Production Line

**Figure 12:**
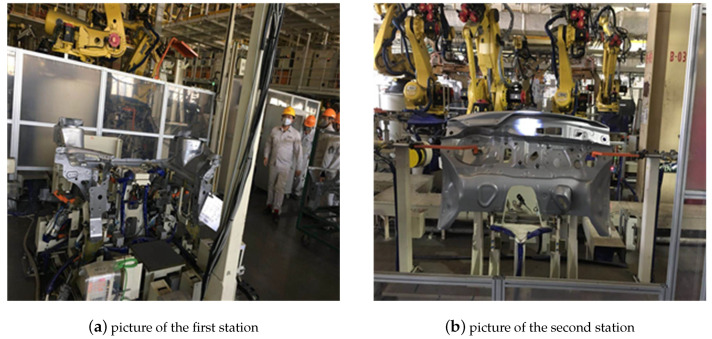
Two different stations in the automobile production line with intelligent grasping system: (**a**) the first station; (**b**) the second station.

On this basis, the vision-based grasping system has been used in automobile intelligent manufacturing by reconfiguring the traditional production line. Through the integration with production line (see Figure 12), production-manufacturing can be accomplished intelligently, quickly and accurately with the grasping system.

To quantitatively evaluate its effectiveness and measurement accuracy of the intelligent system, we conduct a verification experiment on the automobile production line. In this experiment, the vehicle workpieces (floor panel and bonnet in the test) to be operated are fixed on the tooling which is made up of multiple fixtures. Each fixture can be controlled to move forward or backward, up or down. By adjusting the states of all the fixtures, the workpieces can be rotated and translated. The state parameters of all fixtures can be directly read by the tooling controller and transformed into the 6-DOF pose of the workpiece fixed on it. In the experiment, the pose parameters of the tooling are recorded as the ground-truth value $P_{true}$. The intelligent grasping system measures the current workpiece and calculates the position and orientation of the workpiece which is relative to the camera. Then the relative pose is transformed to the end of the welding robot through the external parameters obtained by calibration, so as to guide the welding robot to a specific position accurately and complete the welding operation. The pose parameters measured by the camera are denoted as calculated value $P_{comp}$, so the measurement error of the system is $P_{err}=P_{true}-P_{comp}$.

In the applications, two different stations are selected for measurement. In the first station, there are four cameras, fixed on the upper side (two cameras) and back side (two cameras) of the workpiece. In the second station, there are three cameras, fixed on upper side (one camera) and back side (two cameras) of the workpiece. The measurement error of the two stations is shown in Table 2 and Table 3.

There are 9 sets of measurements in the first station. The average rotation error (°) is (0.20,0.04,−0.05)±(0.29,0.25,0.06), and the average translation error (mm) is (0.24,0.23,0.06)±(0.39,0.25,0.31). Similarly, in the second station there are 15 sets of measurements. The average rotation error (°) is (0.33,−0.05,−0.05)±(0.34,0.21,0.09), and the average translation error (mm) is (0.31,−0.26,0.04)±(0.29,0.37,0.35). From the two Tables (Table 2 and Table 3), we can see that the vision measurement is accurate. What is more, the time consuming of each measurement is less than 50 ms. The system has been applied in actual vehicle production line and the result of long-term operation shows that it can correctly and quickly guide the welding robot arm to complete the welding operation and meet the production requirements of the vehicle production line.

## 4. Conclusions

In this paper, a multi-view-based pose estimation method is proposed, while an intelligent grasping system is established and applied in intelligent manufacturing. This method is designed for production lines with intelligent requirements, capable of measuring zero-offset poses and adjusting the robot path to complete grasping parts. The industrial camera is fixed on the end of the robot. The robot brings the camera to measure the feature points on the parts in different views. This characteristic makes the proposed method applicable to the posture measurement of large-size parts, and can also solve the posture estimation to a certain extent. In addition, this paper proposes a hand-eye calibration method, which can quickly calibrate the transformation relationship between the camera coordinate system and the robot system. Simulation experiments and actual applications have proved the effectiveness and accuracy of the proposed method. It is hoped that the multi-view-based pose estimation method and the established intelligent grasping system can be applied to more intelligent manufacturing scenarios.

## Figures and Tables

**Table 1:** Pose estimation errors (rotation and translation) compared between proposed method and robot teaching (seen as ground-truth).

Index	Rotation Error (Degree)			Translation Error (mm)		
	α	β	γ	X	Y	Z
1	0.31	−0.11	0.06	0.64	−0.68	0.47
2	0.26	−0.23	0.08	0.57	−0.37	−0.11
3	0.29	−0.31	−0.04	0.65	−0.16	−0.14
4	0.17	−0.02	0.03	0.19	−0.62	0.06
5	−0.30	0.03	−0.16	0.27	−0.05	−0.30
6	0.23	0.26	−0.07	0.24	−0.59	−0.51
7	0.10	0.15	−0.09	0.39	0.09	0.07
8	−0.08	−0.34	−0.13	0.12	−0.42	0.43
9	0.30	0.14	−0.11	−0.27	0.44	0.42

**Table 2:** The measurement error of workpiece in the first station.

Index	Rotation Error (Degree)			Translation Error (mm)		
	α	β	γ	X	Y	Z
1	0.61	−0.11	0.06	0.64	−0.68	0.47
2	0.56	−0.23	0.08	0.57	−0.37	−0.11
3	0.69	−0.31	−0.04	0.65	−0.16	−0.14
4	0.37	−0.02	0.03	0.19	−0.62	0.06
5	−0.30	0.03	−0.16	0.27	−0.05	−0.30
6	0.23	0.26	−0.07	0.24	−0.59	−0.51
7	0.60	0.15	−0.09	0.39	0.09	0.07
8	−0.08	−0.34	−0.13	0.12	−0.42	0.43
9	0.30	0.14	−0.11	−0.27	0.44	0.42

**Table 3:** The measurement error of workpiece in the second station.

Index	Rotation Error (Degree)			Translation Error (mm)		
	α	β	γ	X	Y	Z
1	0.42	−0.00	0.02	0.80	−0.25	−0.38
2	−0.07	0.03	0.07	0.37	−0.22	0.17
3	0.19	−0.09	−0.09	0.15	0.45	0.17
4	−0.34	−0.09	−0.01	0.28	0.32	0.39
5	0.39	0.16	−0.11	0.56	0.11	0.01
6	−0.18	−0.21	−0.11	0.47	0.07	0.02
7	0.43	0.31	0.00	0.38	0.35	−0.08
8	0.27	0.24	−0.05	0.13	0.20	0.57
9	0.39	−0.50	−0.01	0.29	0.26	0.18
10	0.34	0.23	−0.02	−0.55	0.47	0.22
11	0.34	0.46	−0.09	0.07	0.42	0.35
12	0.42	−0.25	−0.01	0.74	0.18	−0.51
13	0.41	0.27	−0.14	−0.19	0.73	0.05
14	0.37	0.10	−0.08	0.52	0.11	−0.46
15	−0.39	−0.08	−0.16	−0.43	0.25	0.21
